# Performance evaluation of leukocyte differential on the hematology analyzer Celltac G compared with two hematology analyzers, reference flow cytometry method, and two manual methods

**DOI:** 10.1002/jcla.23827

**Published:** 2021-06-12

**Authors:** Kenji Yamade, Toshihiro Yamaguchi, Yutaka Nagai, Toshinori Kamisako

**Affiliations:** ^1^ Department of Central Clinical Laboratory Kindai University Hospital Osakasayama Japan; ^2^ Kindai University Graduate School of Medical Sciences Osakasayama Japan; ^3^ Faculty of Clinical Laboratory Kansai University of Health Sciences Kumatori Japan; ^4^ IVD Business Operations Nihon Kohden Corp Tokyo Japan

**Keywords:** accuracy, Celltac G, hematology analyzer, leukocyte differential counting, smudge cell

## Abstract

**Background:**

The automated hematology analyzer Celltac G (Nihon Kohden) was designed to improve leukocyte differential performance. Comparison with analyzers using different leukocyte detection principles and differential leukocyte count on wedge film (Wedge‐Diff) shows its clinical utility, and comparison with immunophenotypic leukocyte differential reference method (FCM‐Ref) shows its accuracy performance.

**Methods:**

For method comparison, 598 clinical samples and 46 healthy volunteer samples were selected. The two comparative hematology analyzers (CAAs) used were XN‐9000 (Sysmex) and CELL‐DYN Sapphire (Abbott). The FCM‐Ref provided by the Japanese Society for Laboratory Hematology was selected, and a flow cytometer Navios (Beckman‐Coulter) was used. In manual differential, two kinds of automated slide makers were used: SP‐10 (Sysmex) for wedge technique and SPINNER‐2000 (Lion‐Power) for spinner technique. The spinner technique avoids the issue of Wedge‐Diff smudge cells by removing the risk of breaking cells and non‐uniformity of blood cell distribution on films (Spinner‐Diff).

**Results:**

The Celltac G showed sufficient comparability (*r* = 0.67–1.00) with the CAAs for each leukocyte differential counting value at 0.00–40.87(10^9^/L), and sufficient comparability (*r* = 0.73–0.97) with FCM‐Ref for each leukocyte differential percentage at 0.4–78.5. The identification ratio of the FCM‐Ref in CD45‐positive cells was 99.7% (99.4% to 99.8%). Differences were found between FCM‐Ref/Celltac G/XN‐9000/Spinner‐Diff and Wedge‐Diff for monocytes and neutrophils. The appearance ratio of smudge cells on wedge and spinner film was 12.5% and 0.5%.

**Conclusion:**

The Celltac G hematology analyzer's leukocyte differential showed adequate accuracy compared with the CAAs, FCM‐Ref, and two manual methods and was considered suitable for clinical use.

## Graphical abstract

Leukocyte differential in two hematology analyzers and a manual leukocyte differential method on spinner film showed consistency compared with the reference flow cytometry method provided by an immunophenotypic leukocyte differential reference method, whereas in a manual leukocyte differential method on wedge film for percentages of monocytes and neutrophils showed inconsistency.

Abbreviatons: TAA, Test automated analyzer; CAA: Comparative automated analyzer; BA, basophil; EO, eosinophil; LY, lymphocyte; MO, monocyte; NE, neutrophil; 5diff, number of identified normal five‐part leukocyte WBC cell differential events; CD45+, number of CD45‐positive cell events.

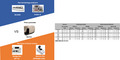

## 1 INTRODUCTION

Different hematology analyzer models use various principles to measure the complete blood count (CBC) and leukocyte differential for routine tests in clinical laboratories. The model‐to‐model measurement dispersion is a known issue in external quality control surveys using fresh blood samples.^1^ Consequently, the accuracy performance of a hematology analyzer is evaluated using the manual differential leukocyte (Manual‐Diff) on blood wedge film (Wedge‐Diff) as the traditional reference method.^2^ However, this method suffers from several disadvantages, including statistical error, slide distribution error, and morphological interpretation error.^3^ The Wedge‐Diff is influenced by non‐uniform distribution, especially of large nucleated cells, on the blood film.^2^ Therefore, these errors should be minimized when evaluating accuracy performance. Elevated numbers of smudge cells tend to be present in the wedge film, especially in case such as chronic lymphocytic leukemia.^4^ The addition of bovine serum albumin (BSA) to blood samples effectively reduces the risk of erroneously generating smudge cells, and it keeps the chromatin structure on wedge film.^5^ An even more effective method to reduce the number of smudge cells on the film is the spinner film, and few smudge cells are found when performing the Manual‐Diff on spinner film (Spinner‐Diff). Hence, the Spinner‐Diff has the potential to improve both the slide distribution error and the morphological interpretation error.^6^ To improve the statistical error, current guidelines^2^, ^7^ recommend using an immunophenotypic leukocyte differential reference method (FCM‐Ref) to verify the leukocyte differential accuracy in normal blood samples. Additionally, the performance of the FCM‐Ref should have an identification ratio of more than 99% of normal leukocyte in CD45‐positive cells to be sufficient in detecting the dispersion and bias, including for small proportion cells such as monocytes and basophils.^1^ The Japanese Society for Laboratory Hematology provided an FCM‐Ref with sufficient performance (JSLH‐Diff) for the present study. This JSLH‐Diff had been assessed^1^ with both the Wedge‐Diff^2^ and the internationally recommended FCM‐Ref.^7^ Hence, the JSLH‐Diff was selected as the FCM‐Ref in this study. When evaluating the accuracy performance of the hematology analyzers’ leukocyte differential, establishing the true quantitative value may be challenging. Therefore, it is desirable to simultaneously compare with FCM‐Ref as a reference method, the Wedge‐Diff as a traditional reference method, and the Spinner‐Diff as an improving Wedge‐Diff. In this study, the clinical usefulness and the accuracy performance of the automated hematology analyzer Celltac G (MEK‐9100; Nihon Kohden) were assessed.

## 2 MATERIALS AND METHODS

The present study was conducted at the Kindai University Hospital (Osakasayama, Japan) using 598 peripheral venous blood samples from hospitalized and ambulatory patients collected during a 4‐month period in 2017. Further, samples from 46 healthy volunteers were also used during a 2‐month period in 2018. The hematology analyzer measurements and FCM‐Ref were conducted within 4 hours of blood collection. Blood films were stained with May‐Giemsa.^2^ The FCM‐Ref was completed within the period during which the prepared samples were stable.^7^ Samples were used after completion of routine testing. This evaluation was carried out according to the International Council for Standardization in Haematology (ICSH) recommendations^7^ and the Clinical and Laboratory Standard Institute (CLSI) guidelines.^2^, ^8^ This study was approved by the institutional review boards (IRB No.: 28–057 ER66‐05). Informed consent was obtained from those who voluntarily agreed to participate in this study and in form of opt out from patients.

### 2.1 Blood samples

All samples were collected in tubes containing K2‐EDTA.^9^ The blood collection tubes,^10^ blood collection procedure,^11^ and mixing procedure^12^ were according to the methods described by ICSH and CLSI. For method comparison between the three analyzers, 388 clinical samples were used. Next, for method comparison between the three analyzers and Manual‐Diff, other 210 clinical samples were used. For accuracy evaluation between FCM‐Ref and two analyzers, 46 normal samples from healthy volunteers were used. Criteria for reference individuals for establishing reference intervals were used to select healthy volunteer donors.^13^ The following occurrences were excluded from sample selection: Failure to adhere to the study‐specific procedure; Instrument, operator‐related, or sample‐related failure; and a data‐invalidating flag as described in the operating instructions for each instrument.^2^

### 2.2 Statistical analysis

Statistical analysis was performed with the following software: Excel 2010 (Microsoft); MedCalc 12.7.8.0 (MedCalc Software); StatFlex ver.7 (Artech); Method Validation version 5.10.9 (Analyze‐it Software). Correlation coefficients were calculated by the least‐square method and the intercept, the slope, and the 95% confidence intervals (95% CI) by Passing‐Bablok regression and Bland‐Altman differential analysis.^14^

### 2.3 Measurement method

#### 2.3.1 Hematology analyzers

The Celltac G equipped with software version 01–12 was used as the test automated analyzer (TAA). The Celltac G measures leukocyte differential using novel swirling sheath flow control technology, DynaHelix flow technology^TM^, and the sample leukocytes largely maintain their morphological characteristics with its novel process for lysing. The DynaScatter laser technology^TM^ classifies by three‐dimensional scattergram using optimized scatter light collection angles, which has shown improvements in the measured cell volume accuracy and cell identification.^15^ The XN‐9000 (Sysmex Corporation) equipped with software version 18.0 was used as a comparative automated analyzer (CAA). The CELL‐DYN Sapphire (Abbott Diagnostics) equipped with software version 4.1 was also used as a CAA.

#### 2.3.2 Flow cytometric reference method for leukocyte differential count

**FIGURE 1:**
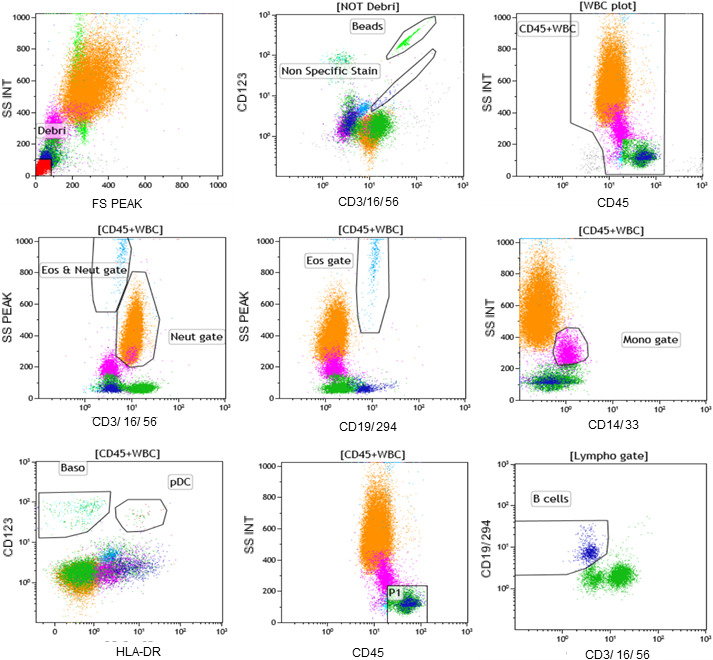
Gating strategy applied to cell type detection of the JSLH‐Diff method. Leukocytes (CD45^+^); lymphocytes (T cells and NK cells CD3^+^CD16^+^CD56^+^/ B cells CD19^+^); neutrophils (CD16^+^); monocytes (CD14^+^CD33^+^); eosinophils (CD294^+^); and basophils (CD123^+^HLA‐DR^−^). Color of each cell cluster: lime (beads), blue and green (lymphocytes), orange (neutrophils), light‐sky‐blue (eosinophils), violet (monocytes), cyan (basophils), red (Debri), and cobalt blue (Non Specific Stain). Antibody reagent: CD45 APC‐H7, CD3/ CD16/ CD56 FITC, CD19/ CD294 APC, CD14/ CD33 PE‐Cy7, CD123 PE, HLA‐DR Per‐CP. APC, allophycocyanin; PE, phycoerythrin; PE‐Cy7(PC7), phycoerythrin ‐cyanin;7, FITC, fluorescein isothiocyanate; PerCP, peridinin chlorophyll protein. BD Trucount^TM^ tubes were used to determine the absolute concentration of the cell populations in addition to their percentages

The JSLH‐Diff was selected as the FCM‐Ref. The JSLH‐Diff was performed using a Navios (Beckman‐Coulter) with the antibody cocktail for JSLH‐Diff (Figure 1)^1^ and carried out according to standard operating procedure (SOP),^16^ the antibody identification checklist,^17^ and using the flowcytometer setting.^18^ Blood samples (50 μl) were stained with the antibodies. Erythrocyte lysis was performed using a no‐wash procedure. The identification ratio of 99% or more was required. This condition was used as an index of the measurement performance validity of the laboratory reference method to test proficiency and to determine whether measurements and analyses were performing well.

#### 2.3.3 Manual reference method for leukocyte differential count

**FIGURE 2:**
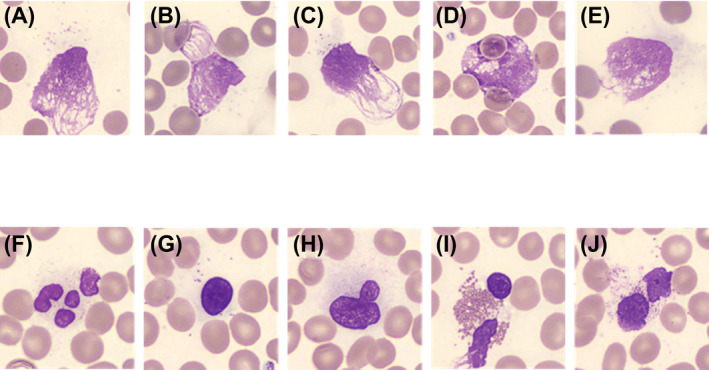
Classification criteria for smudge cells used in this study. The cells were classified into identifiable cells (eg, normal leukocytes and basket cells) and unidentifiable cells, excluding basket cells. Cells lacking cytoplasm are smudge cells(A–J). A basket cell is a smudge cell, which is difficult to distinguish due to the degeneration of karyotype and nuclear structure(A–E). Identified smudge cells (F: neutrophils, G: lymphocytes, H: monocytes, I: eosinophils, and J: basophils) are smudge cells that can be classified by karyotype, nuclear structure, and cytoplasmic granules. Unidentifiable smudge cells are smudge cells that cannot be classified due to its karyotype, nuclear structure, and cytoplasmic granules

Qualified examiners conducted Manual‐Diff identification^2^ and counting.^2^ Blood smears were prepared using both the wedge method,^2^ and the spinning method. The wedge films were prepared by the automated slide maker and stainer Sysmex SP‐10, using the wedge technique. The spinner films were prepared by the slide spinner SPINNER 2000 (Lion Power) using the spinning method. Manual‐Diff was performed on both the wedge film (Wedge‐Diff) and the spinner film (Spinner‐Diff). A DM9600 (Cellavision Japan) was used to clarify the definition of the best reading position by the red blood cell distribution on each film for leukocyte differential. The definition of smudge cells was shown in Figure 2. Images of each cell were acquired using the DM9600 to assess counting in Manual‐Diff. Cell classification, including the number of smudge cells, was performed using the Manual‐Diff methods (Wedge‐Diff and Spinner‐Diff).

### 2.4 Comparability

#### 2.4.1 Comparability with the hematology analyzers

For method comparison between the TAA and the two CAAs, test data were measured using 388 samples. Single measurements were used as the test values for routine tests with the CAAs, and the means of the duplicate measurements were used for confirming the reproducibility by the TAA.

#### 2.4.2 Comparability with Wedge‐Diff in negative samples

For method comparison between Manual‐Diff using wedge blood smear and the three analyzers (TAA and CAAs), test data were measured using 210 samples, and 14 samples with positive findings^19^ on film were excluded.

### 2.5 Accuracy performance in leukocyte normal samples

To clarify the accurate bias differences in normal samples within 1%, 46 normal samples from healthy volunteers were used. Two hematology analyzers (TAA and CAA: XN) and two Manual‐Diff (Wedge‐Diff and Spinner‐Diff) were compared with the JSLH‐Diff as an FCM‐Ref. A regression analysis was performed. Each bias of the mean of all samples to the JSLH‐Diff was calculated.

## 3 RESULTS

### 3.1 Comparability

The results of the comparison between the TAA and the CAAs are shown in Table 1. The results compared with Wedge‐Diff in the TAA and the CAAs are shown in Table 2.

**TABLE 1:** Comparability of Celltac G (TAA) with the measurements of two comparative analyzers (CAA)

		Celltac G			XN−9000					
					Passing‐Bablok					Bland‐Altman
Measurand	Unit	n	Min	Max	Min	Max	r	Intercept (95%CI)	Slope (95%CI)	Bias (95%CI)
WBC	10^9^/L	388	0.24	60.77	0.16	67.59	1.00	−0.03 (−0.07–0.01)	0.96 (0.95–0.96)	−0.35 (−0.40–−0.31)
NE	10^9^/L	388	0.01	40.87	0.02	43.83	1.00	0.01 (−0.02–0.03)	0.96 (0.95–0.96)	−0.20 (−0.25–−0.16)
LY	10^9^/L	388	0.02	11.96	0.01	6.24	0.87	0.02 (−0.01–0.06)	0.94 (0.92–0.96)	−0.06 (−0.11–−0.02)
MO	10^9^/L	388	0.01	37.45	0.00	52.15	0.99	−0.01 (−0.03–0.00)	0.79 (0.76–0.83)	−0.15 (−0.23–−0.08)
EO	10^9^/L	388	0.00	5.45	0.00	4.66	0.74	0.02 (0.01–0.02)	1.11 (1.08–1.14)	0.04 (0.02–0.07)
BA	10^9^/L	388	0.00	1.16	0.00	0.48	0.67	0.01 (0.00–0.01)	1.25 (1.13–1.33)	0.02 (0.02–0.03)
%NE	%	388	0.8	94.8	5.9	98.4	0.97	1.9 (0.6–3.3)	0.98 (0.96–1.00)	0.3 (0.1–0.7)
%LY	%	388	1.0	88.4	0.8	88.2	0.98	0.3 (−0.3–0.8)	1.00 (0.98–1.02)	0.1 (0.2–0.4)
%MO	%	388	0.7	82.3	0.0	78.9	0.92	−0.3 (−0.7–0.1)	0.84 (0.80–0.89)	−1.4 (−1.7–−1.2)
%EO	%	388	0.1	27.5	0.0	44.0	0.76	0.4 (0.4–0.5)	1.10 (1.07–1.13)	0.6 (0.4–0.9)
%BA	%	388	0.1	14.5	0.0	4.50	0.62	0.2 (0.1–0.3)	1.12 (1.02–1.23)	0.4 (0.3–0.5)

		Celltac G			CELL DYN Sapphire					
					Passing‐Bablok					Bland‐Altman
Measurand	Unit	n	Min	Max	Min	Max	r	Intercept (95%CI)	Slope (95%CI)	Bias (95%CI)
WBC	10^9^/L	388	0.24	60.77	0.22	61.62	1.00	−0.03 (−0.07–0.01)	0.99 (0.98–1.00)	−0.12 (−0.15–−0.08)
NE	10^9^/L	388	0.01	40.87	0.02	41.83	0.99	0.02 (0.00–0.05)	0.99 (0.98–1.00)	−0.05 (−0.10–0.00)
LY	10^9^/L	388	0.02	11.96	0.04	31.37	0.87	−0.01 (−0.05–0.02)	0.96 (0.94–0.98)	−0.14 (−0.24–−0.04)
MO	10^9^/L	388	0.01	37.45	0.00	22.00	0.95	−0.01 (−0.03–0.00)	0.85 (0.81–0.89)	0.00 (−0.10–0.11)
EO	10^9^/L	388	0.00	5.45	0.00	7.03	0.92	0.01 (0.00–0.01)	1.21 (1.17–1.25)	0.04 (−0.02–0.06)
BA	10^9^/L	388	0.00	1.16	0.00	0.60	0.75	0.01 (0.00–0.01)	1.81 (1.59–2.02)	0.03 (−0.03–0.04)
%NE	%	388	0.8	94.8	6.0	97.9	0.96	2.2 (0.93–3.54)	0.98 (0.96–1.00)	0.06 (0.2–1.1)
%LY	%	388	1.0	88.4	0.7	89.5	0.96	−1.1 (−1.63–−0.50)	1.01 (0.99–1.03)	−0.9 (−1.3–−0.5)
%MO	%	388	0.7	82.3	0.2	70.0	0.84	0.0 (0.32–0.43)	0.83 (0.79–0.89)	−0.9 (−1.2–−0.5
%EO	%	388	0.1	27.5	0.0	24.6	0.91	0.3 (0.21–0.33)	1.17 (1.13–1.20)	0.6 (−0.5–0.7)
%BA	%	388	0.1	14.5	0.00	2.72	0.16	0.3 (0.21–0.34)	1.42 (1.25–1.62)	0.5 (−0.4–0.6)

Note

A: Comparability of Celltac G (TAA) with the measurements of two CAAs that use different measuring principles: XN‐9000 (Sysmex) and CELL‐DYN Sapphire (Abbott).

Abbreviatons: TAA, Test automated analyzer; BA, basophil; CAA: Comparative automated analyzer; EO, eosinophil; LY, lymphocyte; MO, monocyte; NE, neutrophil; WBC, white blood cell.

**TABLE 2:** Comparability with manual leukocyte differential method on wedge film in negative samples and the three analyzers (TAA and CAAs)

	Manual leukocyte differential				Celltac G			
	wedge film					Passing‐Bablok		Bland‐Altman Bias
Measurand	Sample	n	Min	Max	r	Intercept (95%CI)	Slope (95%CI)	95%CI
%NE	Negative	196	19.8	95.5	0.966	−2.6 (−5.3–−0.5)	1.03 (0.99–1.07)	‐1.1 (−1.6–−0.5)
%LY	Negative	196	0.8	74.8	0.966	−0.8 (−1.8–0.1)	1.05 (1.00–1.09)	0.3 (−0.2–0.8)
%MO	Negative	196	0.0	17.8	0.504	0.7 (0.0–1.3)	0.74 (0.65–0.84)	‐1.1 (−1.5–−0.7)
%EO	Negative	196	0.0	23.3	0.924	0.6 (0.5–0.7)	1.33 (1.23–1.44)	1.2 (1.0–1.4)
%BA	Negative	196	0.0	1.5	0.280	–	–	0.8 (0.7–1.0)

	Manual leukocyte differential				XN−9000			
	wedge film					Passing‐Bablok		Bland‐Altman Bias
Measurand	Sample	n	Min	Max	r	Intercept (95%CI)	Slope (95%CI)	95%CI
%NE	Negative	196	19.8	95.5	0.981	−5.0 (−7.1–−3.1)	1.06 (1.03–1.09)	−1.6 (−2.1–−1.2)
%LY	Negative	196	0.8	74.8	0.976	−0.7 (−1.7–0.0)	1.03 (1.01–1.07)	0.3 (−0.1–0.7)
%MO	Negative	196	0.0	17.8	0.824	0.3 (−0.3–0.9)	1.02 (0.93–1.11)	−0.5 (−0.2–−0.7)
%EO	Negative	196	0.0	23.3	0.947	0.1 (0.0–0.1)	1.25 (1.20–1.33)	0.6 (0.4–0.7)
%BA	Negative	196	0.0	1.5	0.380	–	–	0.5 (0.4–0.6)

	Manual leukocyte differential				Sapphire			
	Wedge film					Passing‐Bablok		Bland‐Altman Bias
Measurand	Sample	n	Min	Max	r	Intercept (95%CI)	Slope (95%CI)	95%CI
%NE	Negative	196	19.8	95.5	0.964	−4.9 (−7.1–−2.8)	1.05 (1.02–1.08)	−2.1 (−2.7–−1.5)
%LY	Negative	196	0.8	74.8	0.967	−0.1 (−1.8–0.7)	1.03 (1.00–1.07)	1.0 (−0.5–1.5)
%MO	Negative	196	0.0	17.8	0.609	0.1 (−0.7–0.7)	1.00 (0.91–1.11)	0.4 (−0.0–−0.8)
%EO	Negative	196	0.0	23.3	0.950	0.3 (0.2–0.3)	1.16 (1.08–1.25)	0.6 (0.4–0.7)
%BA	Negative	196	0.0	1.5	0.366	–	–	0.3 (0.3–0.4)

Note

TAA: Celltac G (Nihon Kohden), CAAs: XN‐9000 (Sysmex) and CELL‐DYN Sapphire (Abbott). Negative: The samples without positive findings^19^ on wedge film.

Abbreviations: TAA, Test automated analyzer; CAA: Comparative automated analyzer; BA, basophil; EO, eosinophil; LY, lymphocyte; MO, monocyte; NE, neutrophil.

### 3.2 Accuracy performance in normal samples

The identification ratio of all identified five‐part leukocyte differential in CD45‐positive cells was 99.7% (99.4% to 99.8%). Table 3 presents the results comparing the FCM‐Ref, TAA, CAA (XN‐9000), Wedge‐Diff, and Spinner‐Diff for leukocyte differential, reporting the regression analysis and the bias of mean. Bias exceeding 1% was demonstrated in Wedge‐Diff for %NE (+2.52%) and %MO (−1.95%), and in CAA for %LY (−1.11%). The mean appearance rate of smudge cells in Wedge‐Diff in 46 samples was 12.3% all smudge cells, 4.1% unidentifiable smudge cells including basket cells, and 1.4% basket cells. The mean appearance rate in Spinner‐Diff was 0.6% all smudge cells, 0.2% unidentifiable smudge cells including basket cells, and 0.1% basket cells. The mean appearance rate of identified smudge cells (neutrophils, lymphocytes, monocytes, eosinophils, and basophils) was as follows: 4.1%, 3.4%, 0.0%, 0.6%, and 0.1% in Wedge‐Diff; 0.3%, 0.0%, 0.0%, 0.0%, and 0.0% in Spinner‐Diff.

**TABLE 3:**
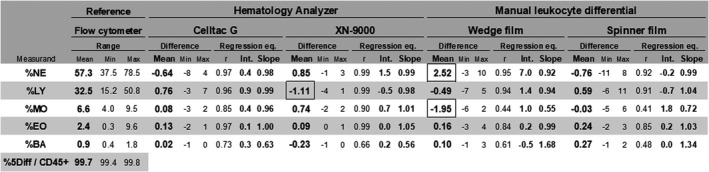
Accuracy performance in leukocyte normal samples

Note

Two hematology analyzers (TAA: Celltac G and CAA: XN‐9000) and two manual leukocyte differential methods on wedge film and spinner film compared with the reference flow cytometry method provided by the Japanese Society for Laboratory Hematology as an immunophenotypic leukocyte differential reference method (Reference). The identification ratio was calculated using the following formula: identification ratio =number of identified normal five‐part leukocyte differential events (5diff) / number of CD45‐positive cell events (CD45+).

Abbreviations: TAA, Test automated analyzer; BA, basophil; CAA: Comparative automated analyzer; EO, eosinophil; LY, lymphocyte; MO, monocyte; NE, neutrophil.

## 4 DISCUSSION

In the present study, the Celltac G demonstrated good comparability with the CAA and the FCM‐Ref and showed acceptable performance for routine use. Specifically, the Celltac G showed sufficient comparability (r=0.67–1.00) with the two hematology analyzers (CAAs) in each leukocyte differential counting value at 0.00–40.87(10^9^/L). The comparison in each leukocyte differential (%) between Wedge‐Diff and the three hematology analyzers (TAA and CAAs) found that the correlation coefficients (*r*) in the negative samples were more than 0.96 for %NE and %LY, 0.92 for %EO, 0.50 for %MO, and 0.28 for %BA. The correlation coefficients in the narrow‐measured ranges and the low ratio leukocyte differentials were low. Regarding the evaluation of the clinical sensitivity for detecting morphologically abnormal cells, 100 or more negative and positive samples each are required,^2^ which will be a subject for subsequent research.

The accuracy performance of the Celltac G compared with the JSLH‐Diff was shown as sufficient in clinical samples. All residuals of the mean values measured by the Celltac G (TAA) compared with the JSLH‐Diff were less than 1%, and the accuracy performance was validated in the TAA for leukocyte differential. In contrast, the bias from the JSLH‐Diff calculated by the mean residual of all samples, which exceeded 1%, was demonstrated in three cases: +2.5% for %NE and −2.0% for %MO in Wedge‐Diff, and −1.1% for %LY in XN (CAA). The Celltac G also includes research parameters, including immature granulocytes, bands, and segment cells, in the differential count. However, this was beyond the scope of the present study as no further information was available. Evaluation of the research parameters should be performed as a next step.

In terms of the FCM‐Ref, all identification ratios of normal nucleated cells in CD45‐positive cells by JSLH‐Diff were 99% or more (0.994–0.998). Therefore, the JSLH‐Diff was determined to be sufficient to verify the inconsistency of the 1% bias. The SOP,^16^ antibody identification checklist,^18^ and FCM^17^ were useful for quality assurance of reference values to set the gate on plots, set the sensitivities, and check the reagent quality.^1^ With this method, the dispersion and bias can be rapidly detected even with small proportion cells (%Mo, % Eo and %Ba) when approximately ten samples are measured and can be used in practice.^1^ In peripheral blood from healthy donors, leukocytes, other than the five‐part leukocyte differential, contain less than 1% of hematopoietic stem cells and dendritic cells.^20^, ^21^ In the JSLH‐Diff, these cells are classified in the lymphocyte fraction of JSLH‐Diff; hence, it was speculated that the <1% unidentified CD45‐positive cells were mainly due to debris.^1^ Regarding the −1.1% bias for %LY in XN (CAA), this may be attributed to significant disruption of the lymphocyte cell membrane by the WDF‐specific reagent used in XN, with almost all cytoplasm being lost. This reagent can also cause a similar loss of intracellular structures as lymphocytes have few organelles.^22^

The effect of non‐uniformity in cell distribution in the blood film in Wedge‐Diff blood film is thought to explain the results obtained in this study for this method (+2.5% for %NE and −2.0% for %MO). The CLSI standard also reported that %MO was 10–20% lower than with the FCM method, including hematology analyzers due to the issue of non‐uniformity.^2^ A tendency was also observed in this study. Additionally, the wide bias observed for %NE was attributed to the small bias for %MO causing wide bias for other cell percentages. The appearance rate of identified smudge cells of neutrophils and lymphocytes was 4.1% and 3.4% in Wedge‐Diff. These traumatic injuries can puzzle morphological evaluation; in addition, unskilled operators can be misled.^23^ The percentage in Manual‐Diff is calculated from identified cells without counting smudge cells, resulting in a leukocyte differential of 100%. These issues should be considered if affected by greater than 1% bias and error.^1^

The leukocyte differential in the hematology analyzers (Celltac G and XN‐9000) and Spinner‐Diff showed consistency compared with JSLH‐Diff. In contrast, inconsistency was observed in Wedge‐Diff for %MO and %NE. The reason is presumed that the Spinner‐Diff was not affected by slide distribution error and morphological interpretation error. In Wedge‐Diff, the presence of smudge cells, even in healthy volunteer's samples, may be one of the factors causing the inconsistency to the FCM‐Ref, the Spinner‐Diff, and the hematology analyzers.

## 5 CONCLUSION

The Celltac G hematology analyzer's leukocyte differential showed adequate accuracy compared with two comparative hematology analyzers, reference flow cytometry method, and two manual methods and was considered suitable for clinical use.

## CONFLICTS OF INTEREST

This work was supported by a grant from Nihon Kohden Corporation. Yutaka Nagai is a current employee of Nihon Kohden Corporation.

## AUTHOR CONTRIBUTIONS

KY, TY, and TK designed the study, analyzed the data, and performed this study. KY wrote the paper. YN researched for all review and writing the paper. All authors declare that they had full access to the data to revise and approve the manuscript.

## Data Availability

The data that support the findings of this study are available from the corresponding author upon reasonable request.
